# A Systematic AQbD Approach for Optimization of the Most Influential Experimental Parameters on Analysis of Fish Spoilage-Related Volatile Amines

**DOI:** 10.3390/foods9091321

**Published:** 2020-09-19

**Authors:** Jorge Freitas, Pedro Silva, Paulo Vaz-Pires, José S. Câmara

**Affiliations:** 1 CQM–Centro Química da Madeira, Campus Universitário da Penteada, 9020-105 Funchal, Portugal; jorgedcfreitas@gmail.com (J.F.); pedro_dasilva@hotmail.com (P.S.); 2 ICBAS–Abel Salazar Institute for the Biomedical Sciences, University of Porto, R. Jorge Viterbo Ferreira, 228, 4050-313 Porto, Portugal; vazpires@icbas.up.pt; 3 CIIMAR–Interdisciplinary Centre of Marine and Environmental Research, Terminal de Cruzeiros de Leixões, Av. General Norton de Matos, S/N, 4450-208 Matosinhos, Portugal; 4 Faculty of Exact Sciences and Engineering, University of Madeira, Campus Universitário da Penteada, 9020-105 Funchal, Portugal

**Keywords:** *Sparus aurata*, trimethylamine, dimethylamine, freshness, HS-SPME, GC-MS, microbiology, dielectric properties, sensory analysis

## Abstract

The volatile amines trimethylamine (TMA) and dimethylamine (DMA) could be used as important spoilage indices for seafood products, assisting in the determination of the rejection period. In the present study, a systematic analytical duality-by-design (AQbD) approach was used as a powerful strategy to optimize the most important experimental parameters of headspace solid-phase microextraction (HS-SPME) and gas chromatography-mass spectrometry (GC-MS) conditions for the quantification of TMA and DMA in *Sparus aurata*. This optimization enabled the selection of the best points in the method operable design region for HS-SPME extraction (30 min; 35 °C; NaOH 15 M and NaCl 35%, *w/v*) and GC-MS analysis (80 °C; gradient 50 °C/min; flow rate 1 mL/min and splitless mode). The rejection day, estimated through the TMA concentration (>12 mg/100 g, at days 9–10), was compared with sensory (quality index method: day 7–8), physical (Torrymeter: day 8–9), and microbial (day 9–10) analysis, corroborating the suitability of the proposed approach for estimating the period for which they will retain an acceptable level of eating quality from a safety and sensory perspective.

## 1. Introduction

Fish constitute a complex system in which enzymatic, microbial, and physicochemical interactions occur simultaneously, which has an impact on flavor, texture, and shelf life (the time a product remains acceptable for consumption). Understanding the shelf life of foods from animal or vegetable origin is of primordial importance in designing appropriate preservation strategies that extend their shelf life [1,2]. After fish death and rigor mortis resolution, a succession of reactions takes place, which are of great importance for fish freshness and shelf life. These reactions are influenced by fish species, their physiological and environmental conditions, (e.g., water temperature), and will impact fish freshness and spoilage progress. Additionally, catching and harvesting methods will have a major influence on fish deterioration. Such alterations can arise from: (i) Chemical deterioration that can be non-enzymatic, enzymatic, or rancidity (oxidative or hydrolytic); (ii) physical changes that can have several forms, such as moisture loss; and (iii) microbiological changes by bacteria, fungi, viruses, and parasites [2,3]. The most common consequences of these processes are pH and physical alterations as well as the production of slime, off-odors, and off-flavors attributed to volatile compounds, mainly amines and short-chain alcohols. Among the amines, the volatile amines trimethylamine (TMA) and dimethylamine (DMA) can be used as potential markers to monitor the spoilage state of fish, assisting in the determination of the time in which the fish will remain acceptable for consumption, retaining its sensory and eating qualities [4,5,6]. The impact of secondary amines on fish products is relevant because of the involvement of amines as probable precursors of carcinogenic compounds.

The most common methods to quantify volatile amines in fish include total volatile base-nitrogen determination (TVB-N) and TMA quantification [7]. The TVB-N measures the ammonia (intrinsic and formed during analysis), TMA, and DMA. The increment in these components, in combination or alone, will increase TVB-N amounts [4,8]. The ammonia present in the immediate postmortem flesh (after death during harvesting or after a few hours in ice storage) has its origin in the inosine monophosphate formed through adenine nucleotide deamination [2,4]. Additionally, trimethylamine oxide demethylase (TMAOase), a molybdoenzyme present in the periplasm of specific spoilage organisms, catalyzes the cleavage of trimethylamine oxide (TMAO) into DMA and formaldehyde (FA). However, several days (±10 days) after the catch, bacterial reduction (genus *Shewanella*, if fish are from cold water, or *Pseudomonad* if fish are from warm water) of TMAO to TMA is the dominant process. During storage, the increment in TMA levels is characterized by a fast increase in the first 5–7 days, followed by an apparent stable concentration. DMA formation, besides not being present in all species, is more relevant during frozen storage even though in small amounts [4,8].

In Europe, chemical analysis of fish samples follows the Regulation (EC) No. 2074/2005, which determines the quantification of TVB-N through steam distillation and establishes acceptable amounts according to fish species [9,10,11]. In the case of the determination of TMA concentration, the official recommended method is based on Dyer’s method [10]. Other common methodologies to quantify the TVB-N, ammonia, TMA, and DMA include steam distillation and titration of the amines, using muscle samples or extracts from the muscle, as described in Table S1 (Supplementary Material). Capillary electrophoresis (CE), high-performance liquid chromatography (HPLC), colorimetry, gas chromatography, flow injection analysis using potentiometric detection, and electronic nose have also been used [8,12].

Even with the automatization and efficiency of recent equipment, several problems are still prevalent and hinder data quality. Recurrent issues, such as decomposition during distillation, incomplete recovery of the amines, interference of the reagents, among others, contribute to bias between results obtained by the different methods, and overestimation of the measured compounds [2,4]. The comprehensive and versatile headspace solid-phase microextraction (HS-SPME), a solvent-free extraction technique that incorporates extraction and concentration in one single step, has demonstrated unique capabilities for the quantitative analysis of volatile components in a wide variety of food samples [13,14,15]. However, as with other methods, there is still a lack of standardization in the extraction protocol, fiber selection, and conditions for GC-MS analysis (Table S2, Supplementary Material).

The analytical quality-by-design (AQbD) approach has been recognized as a reliable methodology for the development of analytical measuring systems and promoted by several international regulators, such as the International Conference on Harmonization [16]. The AQbD principals started on risk management, which initiates with the understanding of the control of the system process and product, through predefined objectives to promote the overall quality [17,18,19]. The application to the analytical methods is supported by the following four key steps: (i) Selection of the analytical target profile (ATP), (ii) definition of a method operable design region (MODR), (iii) integration of risk assessment, and (iv) multivariate statistical analysis. All of them contribute to the overall knowledge of the method and quality verification [17,18,19]. The AQbD concept is commonly applied to pharmaceutical development and was recently introduced in the analysis of foods, reinforcing its contribution to the methods harmonization [20,21,22].

The purpose of this study was to use the AQbD approach as a powerful strategy to determine the optimal analytical conditions for the quantification of TMA and DMA levels in *Sparus aurata* (gilthead seabream, GSB) with origin from aquaculture, by HS-SPME/GC-MS methodology. The optimization approach will allow the selection of the best points in the MODR for HS-SPME and GC-MS analysis. The HS-SPME/GC-MS results will be compared with data from physical, microbial, and sensory analyses. Additionally, the rejection day for the gilthead seabream will be estimated based on the data results from all the analyses.

## 2. Materials and Methods

### 2.1. Reagents and Materials

The amines TMA, DMA, and the internal standard, n-propylamine hydrochlorides, were acquired from BDH and Merck. Sodium chloride (NaCl, 99.5%) and sodium hydroxide (NaOH) were obtained from PanReac (Barcelona, Spain). Ultra-pure water was obtained from a Milli-Q^®^ system (Millipore, Burlington, MA, USA), and helium of purity 5.0 was used as the GC carrier gas, supplied by Air Liquide (Alges, Portugal). The SPME holder for the manual sampling of SPME fiber and the respective fibers, namely the divinylbenzene/carboxen/polydimethylsiloxane (DVB/CAR/PDMS), with a 50/30 mm film thickness, carboxen/polydimethylsiloxane (CAR/PDMS) with a 75 mm film thickness, and polydimethylsiloxane/divinylbenzene (PDMS/DVB) with a 65 mm film thickness, were purchased from Supelco (Bellefonte, PA, USA). Ringer solution (Oxoid, Basingstoke, Hampshire, UK) was used for microorganism transference. Inoculations were made in iron agar, nutrient agar (Oxoid, Basingstoke, Hampshire, UK), and MacConkey agar (PanReac AppliChem, Darmstadt, Germany). For physical evaluation, a Torrymeter 295 (Distell, West Lothian, Scotland, UK) was used.

### 2.2. Degradation Trial

Fresh GSB specimens were kindly given by Ilhapeixe SA facilities, Madeira Island, Portugal. Forty-five specimens were killed in a mix of saltwater and ice using the current company practice. The fish were transported to the company refrigerated facilities 2–3 h after capture. Before storage, the fish were washed with fresh water, then placed in perforated plastic boxes with crushed ice and stored at 0 °C (±1 °C) for 20 days as a maximum. When necessary, ice was added. The sampling periodicity for all experiments were: amines analysis: 0, 4, 8, 12, and 15 days; physical properties: 0–6, 8–10, 12–15, 17, and 20 days; sensory analysis: 0, 1, 3, 5, 7, 9, 12, and 15 days; and microbial analysis: 0, 2, 3, 5, 6, 7, 9, 10, and 15 days.

### 2.3. Sample Preparation for Amine Analysis

Stock solutions of TMA and DMA (1000 µg/mL) were prepared by dissolving quantitative amounts of neat standards in trichloroacetic acid solution (TCA 7.5%). Dilutions of the stock were made to six concentration levels between 2 and 20 µg/mL. Six-point (including blank) calibration curves were established.

Sample preparation for TMA and DMA extraction was based on previous studies [12,23,24]. The fish were filleted, and 10 g of flesh were blended in a solution of TCA 7.5%, with a proportion of 1:2 (*w/w*). The blended fillets were centrifuged at 10,000× *g* for 10 min at 4 °C, and the supernatant was collected and stored at −80 °C until analysis.

### 2.4. HS-SPME Procedure

The HS-SPME procedure was carried out according to Freitas et al. (2019) [24]. In brief, in an 8-mL glass vial, an aliquot of 0.5 mL of the sample supernatant, 1 mL of 15 M NaOH, and 1 mL of NaCl solution (35%, *w/v*) were added. The DVB/CAR/PDMS fiber was placed into the vial headspace at 35 °C (thermostatic bath) for 30 min. After the extraction, the fiber was withdrawn and introduced into the GC injector port at 235 °C for 6 min for the thermal desorption of the analytes. All assays were carried out in triplicates.

### 2.5. GC–MS Conditions

Chromatographic separation was performed using an Agilent 6890N (Palo Alto, CA, USA) gas chromatography system combined with an Agilent 5975 quadrupole mass selective detector and splitless injector equipped with a BP-20 (30 m × 0.25 mm i.d. × 0.25 µm film thickness) fused silica capillary column supplied provided by SGE (Darmstadt, Germany) with helium (Helium N60, Air Liquid, Alges, Portugal) as the carrier gas at a flow rate of 1 mL/min (column-head pressure: 13 psi). An insert of 0.75 mm i.d. was used and the injector temperature was fixed at 235 °C. The GC oven temperature was held for 1 min at 80 °C, and ramped to 220 °C at 50 °C/min (held for 5 min). The manifold, GC-MS interface, and quadrupole temperatures were held at 180, 220, and 180 °C, respectively. In addition, MS detection was performed in full scan, the ion energy used for the electron impact (EI) was 70 eV, and the source temperature was 180 °C. The electron multiplier was set to the auto-tune procedure. The mass acquisition range, made in full scan mode, was 30–300 m/z, 1.9 spectra/s. The TMA and DMA identification was carried out by comparison with the mass spectra and retention times of pure standards using the same instrumental conditions, and by matching the mass spectra with the data system library (NIST05).

### 2.6. Method Validation

The developed and optimized HS-SPME method was properly validated in terms of linearity, detection and quantification limits, intraday and interday precision, accuracy, and matrix effects (ME), according to the Association of Official Analytical Chemists (AOAC) and International Union of Pure and Applied Chemistry (IUPAC) guidelines [25,26]. Matrix-matched calibration curves were obtained for each compound to evaluate the linearity of the proposed method, using six concentration levels for TMA (2, 5, 8, 10, 15, and 20 µg/mL) and seven for DMA (2, 5, 7, 8, 10, 15 and 20 µg/mL). The calibration curves were constructed by plotting the peak area of TMA and DMA against the analyte concentration and were fitted by linear regression analysis. The ME was evaluated by comparing the slopes of the matrix-matched and solvent-based standard calibration curves. Intermediate precision and repeatability were calculated by the standard addition method at a low (3 µg/mL, LL), medium (9 µg/mL, ML), and high concentration level (15 µg/mL, HL) through inter and intraday variation measurements. Recovery was also calculated by spiking a known concentration sample with the previous LL, ML, and HL concentrations. The method limit of detection (LOD) and quantification (LOQ) for each analyte was determined using the residual standard deviation (*Sy/x*) of the corresponding calibration curve. The LOD and LOQ were calculated as 3.3 × ((*Sy/x*)/b) and 10 × ((*Sy/x*)/b), respectively, where (*Sy/x*) represents the standard deviation of the ordinate at origin and *b* represents the slope of the regression line [27].

### 2.7. Shelf Life Estimation

The results were compared with other methods commonly used for shelf life estimation studies [28,29,30,31]. The rejection day obtained with the proposed chromatographic method was compared with results obtained from the sensory analysis by the quality index method (QIM) approach, Torrymeter evaluation for dielectric property measurement, and microbial growth analysis. The microbial experiment was performed as described previously [32]. In summary, the fish skin was swabbed with sterile cotton swabs and the microorganisms were transferred to 1 mL of ringer solution for serial dilutions. Plate inoculation was executed with the 20-μL drop method. Total viable colony counts (TVCcs) were performed after 48 h at room temperature, in the plates of nutrient agar. Iron agar was used for counting H_2_S-producing bacteria after 48 h at room temperature. *Enterobacteriaceae* counts were grown on MacConkey agar and counted after 72 h at 30 °C. In all cases, the values were expressed as colony forming units (cfu). The physical analysis was made using a Torrymeter [24] and measurements were taken on the fish dorsal region as recommended [33]. Sensory evaluation was performed following the QIM protocol, as previously described [34]. In brief, a total of 3 sessions were performed, with two training sessions before to the final experimental session. Six trained assessors participated in the trials and their performance was evaluated following the r^2^ values and the slopes of the quality index (QI) values for each one [24]. The values obtained by each assessor were averaged and summed to express the overall QI. The utilized table for sensory evaluation is presented in Table S3 (Supplementary Material).

### 2.8. Statistical Analysis

Statistical analysis was carried out using the software STATISTICA 10.0 (Stat Soft, Inc., Tulsa, OK, USA), and the significance was set at a probability of *p*-values < 0.05.

## 3. Results and Discussion

### 3.1. Establishing the Quality by Design Bases for Analysis

#### 3.1.1. Defining the Analytical Target Profile and Method Scouting

A relevant step in the AQbD approach is the definition of the analytical target profile (ATP), defined as the separation, identification, and quantification of TMA/DMA in fish muscle extract using HS-SPME/GC-MS analytical methodology.

The scouting of the analytical method, which implies the study of the relevant parameters for the chosen methodology and the target analyte characteristics, was based on previously developed studies for HS-SPME/GC-MS [4,8,12,23,27,35,36,37,38]. The sample preparation was based on the work of Dehaut (2016) to facilitate comparison with other quantification methods [23]. The knowledge from these studies also helped to define. The critical quality attributes, critical process parameters, and quality risk assessment were also defined.

#### 3.1.2. Method Critical Quality Attribute Definition Methods

For the selection of HS-SPME critical method attributes (CMAs), the efficiency of the extraction method was taken into consideration. Therefore, the attributes selected as being important for extraction efficiency include the number of extracted amines, total peak area (TPA), and intermediate precision (IP) of the analytes. TPA, IP, peak resolution (PR), tailing factor (TF), and symmetry factor (SF) of the analytes were the attributes considered for GC-MS analysis.

The requirements for each CMAs were defined as: The amines under analysis must be identified (TMA and DMA); the TPA value will be selected by the maximum that can be reached; and PR values should be greater than 1.5, TF should be lower than 1.5, while SF should be lower than 2.5. For IP, the residual standard deviation percentage values (%RSD) should be as low as possible and are considered acceptable when they are not higher than 15% [25,26].

#### 3.1.3. Critical Method Parameters and Quality Risk Assessment

The selection of the critical method parameters (CMPs) was based on the extraction efficiency of SPME fiber and the GC-MS analytical performance. The selected parameters for quality risk assessment (QRA) are presented through the Ishikawa diagram (Figure S1, Supplementary Material) and were based on scouting phase analysis. For HS-SPME, the chosen CMPs were fiber type, extraction time, extraction temperature, pH (alkalizing agent), and ionic strength (salt concentration, NaCl). The main parameter regarding HS-SPME effectiveness is the fiber type, which is greatly influenced by the selectivity of the polymer that forms the fiber and its interaction with the target analytes [39].

In GC methods, the type of column is a relevant parameter that influences the separation performance. Optimization is achieved by selecting the column constituents according to the functional groups, bonding type, and chemistry of the stationary phase. The other parameters that can be adjusted are gas flow, initial oven temperature, ramp temperature, split method, eluent composition, and EI mode [37,38]. For GC-MS methodology analysis, the chosen critical method parameters were gas flow, initial oven temperature, and ramp temperature. The parameters that were not chosen for investigation were fixed as in previous works [27].

### 3.2. Method Knowledge of Space

#### 3.2.1. HS-SPME Fibers

**Figure 1:**
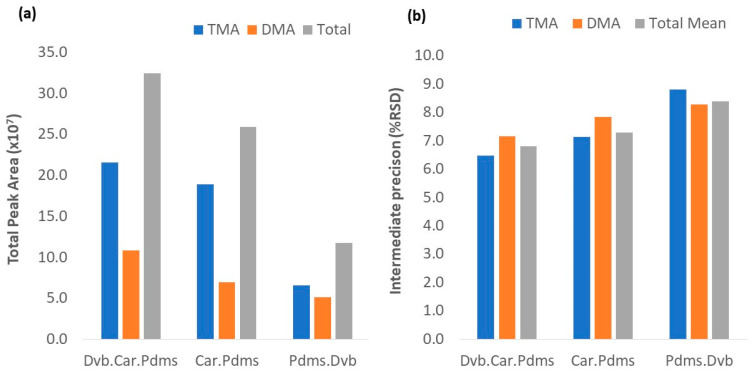
Results from the HS-SPME knowledge space of extraction conditions (**a**) Total peak area analysis for all fibers; (**b**) Intermediate precision for all assayed fibers.

The HS-SPME/GC-MS methods previously described by our and other research groups provided the necessary knowledge for defining the CMPs range, before the investigation of the knowledge space (KS) [12,23,24]. Taking into account the obtained results, a fractional factorial design (FFD) based on a 4^3−1^-level model was applied as the design of the experiment (DoE) for HS-SPME-KS screening, being evaluated by the following parameters: NaOH concentration (5, 15, and 25 M), extraction temperature (25, 35, and 45 °C), extraction time (5, 25, and 45 min), and NaCl percentage (10%, 30%, and 50%). DoE was used for each of the studied fiber types (DVB/CAR/PDMS, CAR/PDMS, and PDMS/DVB). The results are presented in Table S4 (Supplementary Material). The TPA and IP results for each fiber under the optimal extraction conditions are shown in Figure 1(a) and (b), respectively.

According to the results, all fibers were capable of extracting TMA and DMA, which is in agreement with the results obtained by Chan (2006) [12]. However, they showed variable extraction efficiency. The efficiency of the fibers for TMA and DMA extraction followed the order DVB/CAR/PDMS > CAR/PDMS > PDMS/DVB. From the obtained data, CAR/PDMS and PDMS/DVB fibers showed better results when the extraction parameters were in their higher range conditions corresponding to runs 24 and 22, respectively. The DVB/CAR/PDMS fiber presented the best results under milder extraction conditions (run 13), mainly in the extraction time, which was half of the time necessary for the other fibers. At their highest TPA values, the percentage of difference concerning DVB/CAR/PDMS fiber is 20% for CAR/PDMS and 64% for PDMS/DVB. For IP (%RSD), the total means for each fiber performed below the maximum acceptable value of 15% RSD, as presented in Table S4 (Supplementary Material). Only two conditions registered values in the limits of the maximum acceptable value, 15.0% for the DMA value with DVB/CAR/PDMS (run 18), and 15.4% for the TMA value with PDMS/DVB (run 16). These results reflect the fiber properties and amine characteristics as summarized in Table 1.

Four parameters must be taken into consideration for fiber selection:

1. Molecular weight and analyte size related to analyte speed; the smaller the analytes, the faster it moves, and the retention might be harder. Highly cross-linked fibers could better retain fast-moving analytes.
2. Analyte polarity is defined by the type of coating and its ability to retain analytes according to polarity. However, each fiber has a certain capacity that might be better suited for ones than others.
3. Analyte concentration level and range-thicker fibers might require higher concentrations of an analyte to be more efficient.
4. The complexity of the sample-related to the analyte displacement by the other sample components with higher affinity or concentration.

Considering the properties of the fiber, the thicker ones will require more time to reach the equilibrium and a high analyte concentration present in the headspace, and consequently harder extraction conditions (i.e., temperature, extraction time, alkaline conditions, and ionic strength (salt addition). Pore size also influences the compound molecular weight range and its capability to be retained. Thicker coatings tend to be more efficient; however, longer times are required to achieve equilibrium and to analyte desorption. On the other hand, the efficiency of the fiber increases with the decrease in film thickness [37,38,39]. PDMS/DVB showed the lowest extraction efficiency, expressed by the lowest amount of extracted TMA and DMA. Additionally, its lower cross-linked structure decreases the extraction efficiency of low molecular and highly volatile analytes, such as TMA and DMA [37,39]. Even though the CAR/PDMS fiber has a wider range of MW, its low thickness requires a longer extraction time to reach equilibrium and a higher desorption time owing to the coating properties and configuration. Higher temperatures are also required due to their low thickness as well as a higher analyte concentration to improve efficiency [37,39]. DVB/CAR/PDMS fiber has a highly cross-linked structure, an intermediate polarity strength, and a thinner coating layer when compared with PDMS/DVB and CAR/PDMS [37]. Considering the sample complexity, DVB/CAR/PDMS is the most suitable for complex samples, and a low concentration of the target compounds, owing to its mixed layer properties. The largest compounds are retained in the first layer (DVB), maintaining free coating to retain the lowest MW compounds. Therefore, for the next steps, the DVB/CAR/PDMS fiber was chosen because of its overall performance and acceptable values.

#### 3.2.2. GC-MS Conditions

In the GC methodology, some parameters, including the column, splitless mode, flow rate of the carrier gas, and temperature gradient, must be taken into consideration [37,38]. There are several columns for GC analysis and all of them can be suited for numerous analyses; however, it is necessary to evaluate the operational range of the parameters that originate the best possible results. There are few studies on the optimization of GC-MS conditions for volatile amines. The obtained results are presented in Supplementary Material Table S2. Chan (2006) [12] presented a more complete study but with a high emphasis on the SPME fiber selection using a unidimensional approach. On the other hand, Dehaute (2012) [23] studied the impact of some GC-MS conditions on TMA and DMA analysis, such as the injector temperature, oven temperature, and split ratio. In the present study, and according to the obtained results, it was decided to proceed the investigation with a BP-20 (polar) capillary column, an oven temperature ranging between 35 and 85 °C, ramp speed 40–60 °C/min, and a flowrate between 0.8 and 1.3 L/min. The fixed parameters are described in Section 2.4, where the GC-MS conditions are detailed.

### 3.3. Determination of Method Operable Design Region

An MODR was defined for HS-SPME and GC-MS to define the space where the CMPs will fit the purpose of promoting the CMAs responses.

#### 3.3.1. HS-SPME Extraction

The definition of MODR for HS-SPME_(DVB/CAR/PDMS)_ was performed based on CMAs responses (TPA and IP) from interactions between extracted amines. The CMPs analyzed included NaOH concentration, extraction temperature, extraction time, and ionic strength (salt percentage). Figure S2 (Supplementary Material) shows the Pareto chart analysis of the effects between the studied CMPs on the TPA and IP responses. According to the results, the principal factor affecting TPA is the NaOH concentration, while for IP, it is mainly affected by temperature. Alkalinization of the sample will increase the amines volatility and therefore their concentration in the headspace. This is influenced by the pKa value of the amines (Table 1). The NaOH concentration should raise the pH in two units above the pka of the analyte [37]. This effect was confirmed by Dehaut (2016), who verified the appearance of amines after the addition of an alkaline agent [23]. The time of extraction and temperature effects have a role in the development of the equilibrium between the gas phase and the sample. Temperature influences the mass transference kinetics between the sample, headspace, and fiber, and consequently the extraction efficiency and precision [38]. In the case of extraction time, its role is to assure that the amount of the extracted analyte is proportional to the concentration present in the sample [38].

**Figure 2:**
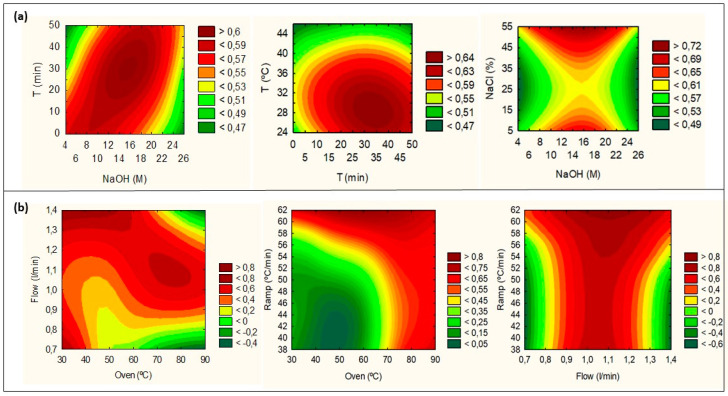
The effects of all CMPs through the desirability surface contour method. (**a**) The desirability plots for HS-SPME(DVB/CAR/PDMS), and simultaneous analysis of the TPA and IP results. (**b**) The desirability plots for CG-MS(BP20), and simultaneous analysis of the TPA, IP, PR, TF, and SF results.

The effects of all CMPs on TPA and IP, respectively, through the desirability surface contour method, are shown in Figure S3a,b (Supplementary Material). The values chosen for TPA to perform this analysis were defined as the highest desirable (3.24 × 10^8^), acceptable mean (1.83 × 10^8^), and lowest unacceptable (4.23 × 10^7^). In the case of IP, the desirability parameters were defined as the highest desirable (1.5), acceptable mean (6.5), and unacceptable (12). Here, it is possible to see that the regions with higher desirable TPA values are between 10 and 24 M for the NaOH concentration, 20 and 50 min for the extraction time, extraction temperature between 25 and 40 °C, and NaCl percentage ranging from 15–45%. In the case of IP, for the same interactions, the lowest desirable RSD values are between 1 and 30 min for the extraction time, 6 and 20 M for the NaOH concentration, 25 and 35 °C for the extraction temperature, and 45% and 55% for the NaCl percentage. Overlaying the CMAs responses (Figure 2a), the desirability plots show a mix between TPA and IP results, resulting in an extended range red area, mostly for the interaction between the NaOH concentration and extraction time. The highest desirability index (dark red zone) is achieved between all CMPs in the following ranges: 10–18 M NaOH concentration; 20–40 min extraction time; 25–35 °C extraction temperature; and 35–55% NaCl percentage.

Data verification was performed through the analysis of the agreement between the predicted and the observed values at the optimal condition range, which are described in Supplementary Material Table S5. According to the results, it was found that all the values observed in the experiments were close to the predicted values. However, the results showed slight differences between the observed and predicted values in CMA responses. Such differences might be due to the extended range values for the CMPs and small variations on the optimum point, which produce variability in the CMA responses. Therefore, the parameters at the optimal point were studied through a robustness test (see Section 3.4).

#### 3.3.2. Chromatographic Conditions

The CG-MS(BP20)-MODR definition was performed based on CMA responses from the interactions between the oven temperature (35, 60, and 85 °C), ramp speed (40, 50, and 60 °C/min), and flow rate (0.8, 1.0, and 1.3 L/min). A 3^3−1^ level FFD was used to perform this study (Supplementary Material Table S6). The Pareto ranking analysis for TPA, IP, PR, TF, and SF is shown in Figure S4 (Supplementary Material), respectively. None of the CMPs had a significant influence on the CMA response. A desirability plot was performed overlaying all CMAs, with the following values being defined for each one (respectively TPA, RSD, PR, TF, and SF): desirable (3.68 × 10^8^, 1.0, 10, 1.0, and 1.0), acceptable (2.0 × 10^8^, 10.0, 5.0, 0.9, and 0.9), and unacceptable (3.20 × 10^7^, 14.0, 2.0, 0.5, and 0.5). The desirability plots of the interaction between the change level of continuous CMPs (flow, ramp, and oven temperature) on CMA responses are shown in Figure 2b, respectively. From the results, it was possible to verify that the higher CMA values were influenced by the flow rate and oven temperature, with two clear desirable areas.

One in the lower temperature range (30–40 °C) is associated with higher flow rates (1.2–1.4 L/min), and the other at a higher temperature range (70–80 °C) is associated with mild flow rates (1.0–1.1 L/min). Ramp temperature did not influence the CMAs much. The ramp temperature vs. flow rate plot shows a clear area between 1.0 and 1.1 L/min, throughout the ramp temperature range. Similar results are presented in the ramp vs. oven temperatures, with the highest desirability between 75 and 90 °C, throughout the ramp temperature range. Taking into consideration the equipment and material consumption, the optimum point for GS-MS(BP20)-MODR was defined with an oven temperature of 80 °C, a flow rate of 1.0 L/min, and a ramp temperature of 50 °C/min. Data verification was performed through analysis of the agreement between the predicted and observed values, which are described in Table S7 (Supplementary Material. According to the results, it was found that all the values observed in the experiments were close to the predicted values. However, the results showed slight differences between the observed and the predicted values in the CMA responses. Such differences might be due to the extended range values for the CMPs and small variations on the optimum point, which produced variability in the CMA responses. Therefore, the parameters at the optimal point were studied through a robustness test (see Section 3.4.).

### 3.4. Robustness and Method Control

Robustness was evaluated at the optimal conditions in the MODR using a new experimental design with CMP range values around the determined optimal value. The results for the CMA and CMP response are described in Tables S8 and S9 (Supplementary Material) for HS-SPME and GC-MS, respectively.

#### 3.4.1. Amines Extraction

**Figure 3:**
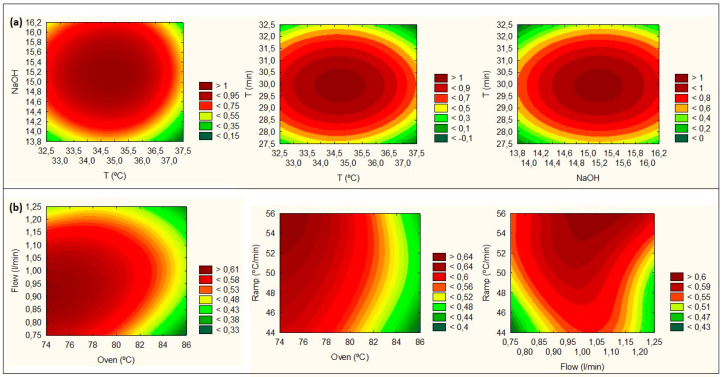
Robustness analysis results of all CMPs through the desirability plots. (**a**) Desirability analysis results for HS-SPME(DVB/CAR/PDMS), simultaneous analysis of TPA, and IP robustness results. (**b**) The desirability analysis for GC-MS(BP20), simultaneous analysis of TPA, IP, PR, TF, and SF robustness results.

The FFD for the robustness screening was based on the 3^3−1^-level factors model, with the temperature (33, 35, and 37 °C), NaOH solution (14, 15, and 16 M), and time (28, 30, and 32 min) being evaluated. The selection of the desirability values was based on the TPA and IP (%RSD) results at the optimum point; therefore, 1% was defined as desirable (7.04 × 10^8^ and 1.68), 0.5% as acceptable (6.12 × 10^8^ and 6.32), and 0% as unacceptable (5.20 × 10^8^ and 10.97). Figure 3a presents the desirability plot for the evaluated conditions. The highest desirability index was reached within the studied range. Table S10 (Supplementary Material) describes the analysis of the agreement between the observed and the predicted values for robustness verification. According to the results, all the observed values are within the range of the predicted values at the 95% confidence interval.

#### 3.4.2. Chromatographic Analysis

The FFD for GC-MS robustness screening was based on the 3^3−1^-level factors model, around the optimal point, with the gas flow rate (0.8, 1.0, and 1.2 L/min), oven temperature (75, 80, and 85 °C), and ramp gradient (45, 50, and 55 °C/min) being evaluated (Table S9, Supplementary Material). A desirability plot was performed by overlaying all CMPs. For the selection of values to be used in the desirability analysis, the TPA, RSD, PR, TF, and SF data were considered at the optimum point, where 1% was defined as desirable (7.5 × 10^8^, 2.0, 3.0, 1.0, 1.0), 0.5% as acceptable (6.0 × 10^8^, 7.0, 2.0, 0.6, 2.3), and 0% as unacceptable (5.2 × 10^8^, 10.0, 2, 0.5, 2.9). The desirability plot is shown in Figure 3b. The highest desirability index was reached with flow rates between 0.9 and 1.1 L/min, oven temperatures of 75–80 °C, and ramp temperature of 50–55 °C/min. In Table S11 (Supplementary Material), the agreement analysis between the predicted and the observed values for robustness verification is shown. From the results, it is possible to verify that all the observed values are within the range of the predicted values, within a confidence interval of 95%, confirming the good robustness of the method. However, the sensitivity of the CMPs influences the robustness analysis, thus it is necessary to establish the system suitability limits (see Section 3.4.3).

#### 3.4.3. Method Control

**Figure 4:**
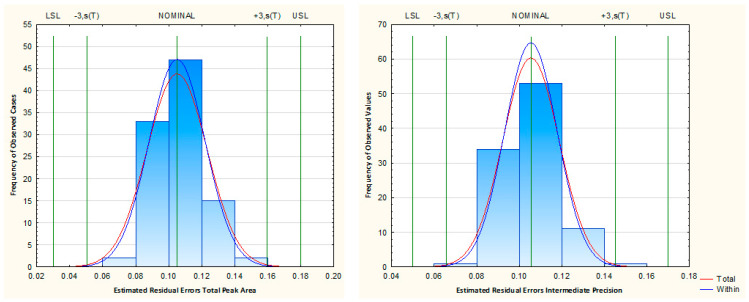
Established limits and results of the capability analysis, for total peak area and intermediate precision.

The method control was performed by generating a large amount of data (100 cases) through Monte Carlo simulation at the CMP range with the highest desirability index. Then, the capability analysis process was applied to the estimated residual errors from all CMA responses. The established limits and results of the capability analysis are listed in Table 2 and presented in Figure 4 for HS-SPME and Figure S5 (Supplementary Material) for GC-MS.

The process capability index (Cpk) values varied between 1.36 to 1.49. From the results, it can be concluded with a high level of confidence that the proposed analytical method is robust, since all CpK values were above the minimal reference value (1.33), for a method to be considered robust [16].

### 3.5. Analytical Method Validation

The method was validated in terms of the linearity of the calibration function, accuracy, precision, limits of detection (LOD), limits of quantitation (LOQ), and ME. Briefly, six TMA and DMA concentrations points were used (2–20 µg/mL range). Linearity was evaluated by the suitability of the function model, comparing the experimental and theoretical Fisher value at 95%. If the dataset expressed a linear function, the condition F_theo_ > F_exp_ is fulfilled [40]. In this work, the described condition was achieved for both amines with a polynomial equation of second degree. The ME was studied using the standard additions method. Repeatability and intermediated precision were used for precision determination, through inter-and intraday variation measurements.

LOQ and LOD were calculated through the regression curve slope and standard deviation of the interception. Table 3 summarizes the obtained results for all the evaluated parameters and confirms that the results are under the stipulated values of the guidelines (ME: 70–125%; intermediate precision and repeatability under 15% RSD; recovery: 80–115%) [25,26].

**Figure 5:**
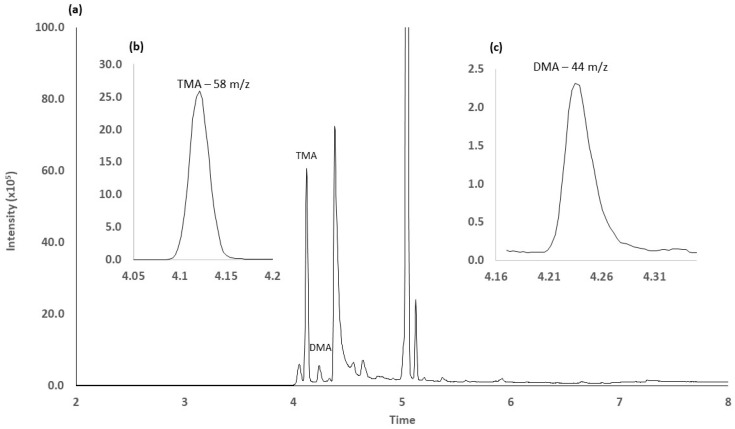
Chromatographic profile of targeted amines with the optimal method conditions (**a**) total ion chromatogram results; (**b**) TMA ion selection 58 m/z. (**c**) DMA ion selection 44 m/z.

Figure 5 shows the chromatographic profile of targeted amines obtained with the optimal method conditions.

### 3.6. Method Application and Shelf Life Estimation

**Figure 6:**
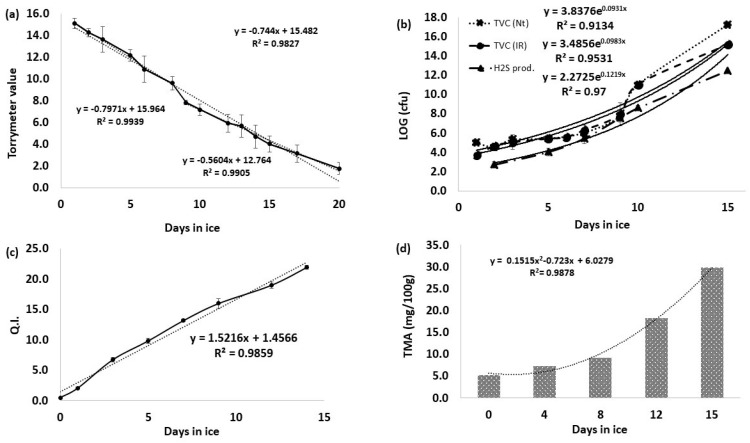
Degradation results in GSB specimens. (**a**) Torrymeter analysis; (**b**) microbial analysis; (**c**) Quality index method analysis; (**d**) TMA chemical analysis with the proposed HS-SPME(DVB/CAR/PDMS)/CG-MS (BP20) method.

To confirm the applicability of the developed method, a degradation experiment with GSB specimens was performed. The deterioration process of fish includes several simultaneous alterations; therefore, different evaluation methods were applied. Besides the proposed chemical analysis, the following methods were applied: dielectric properties with the Torrymeter; sensory analysis following the QIM method; and microbiological analysis through TVCc. The results obtained with each method are presented in Figure 6. With the Torrymeter, the alterations of the muscle texture of GSB were registered throughout the storage experiment. The values started at a maximum value of 15, representing the fresh fish condition. With the progression of the days, the registered data gradually decreased until the value of 8, between 8 and 9 days, at the same time that off-odors were detected (Figure 6a). Other works also propose that when a value of 8 is achieved, the product reaches the borderline for consumption acceptability [24,41,42].

The common initial values for TVCc varies between 10^1^ and 10^4^ (cfu), at first days of storage. At the rejection time, the TVCc values could be between 10^7^ and 10^9^ (cfu), depending on fish species [32,43], as in this case, as the TVCc limit was achieved at 8–9 days (Figure 6b). For *Enterobactereaces* counts, the values throughout the experiment never exceeded 10^2^ (cfu), indicating appropriate hygiene and handling conditions of the product. Similar results were also described in other works [44].

Application of the QIM methodology allowed to determine altered GSB freshness levels. Through the analysis of QIM data (Figure 6c), the odor was a lead contributor to evaluate the degradation progress. During the three first days, gills and skin odor were described as being similar to seaweed or sea breeze. From the third day, the neutral descriptor was used to describe the condition in which the specimens had no clear distinctive odor. Degradation began to be perceived on days 5–6, with off-odor emergence but was only considered significant for rejection from day 7–8, associated with rancid odors (Table S12, Supplementary Material).

According to the European Directive 91/493/EEC, TMA presence in samples is limited to 12 mg/100 g [45]. From the chromatographic results (Figure 6d), it is seen that after the slow increase of the TMA, the limit is achieved between 8 and 12 days. Applying the fitted model, the TMA limit could be achieved between 9 and 10 days. Regarding the DMA analysis, even though the methodology allows simultaneous extraction and quantification, in the present study, the DMA was detected, but its values during the storage remained residual in comparison with TMA. Table 4 compiles the information obtained from the different methods, showing the association between the different used methods to predict and estimate GSB shelf life.

## 4. Conclusions

A systematic AQbD approach was used to improve the knowledge around the HS-SPME/GC-MS method for TMA and DMA analysis in fish samples. After the extraction optimization, the proposed conditions for its implementation were based on the use of DVB/CAR/PDMS fiber, with 0.5 mL of the sample, 1 mL of the NaOH solution (15 M), and 1 mL of the NaCl solution (35%), in a water bath at 35 °C for 30 min. For GC-MS analysis, the best instrumental conditions achieved with a BP-20 column were an oven temperature of 80 °C, ramp speed of 50 °C/min to a maximum of 220 °C, and gas flow rate (helium) of 1.0 mL/min. The method robustness was demonstrated through the Monte Carlo simulation and capability analysis process (1.36–1.49 Cpk). The method validation revealed the satisfactory figures of merit, demonstrating its suitability for the target analysis (Table 3). The method was tested on GSB specimens through the simulation of a degradation experiment, using complementary methodologies to confirm the shelf life results, such as microbial analysis, sensory analysis, and physical analysis. The outcome allows the proposal that the criteria of rejection through TMA analysis (12 mg/100g), was reached between 9 and 10 days. Additionally, analyzing all the results, it is possible to suggest a shelf life period of 9 days (±1 day) for GSB, under the specific conditions of the present study. Besides, not all fish species generate TMA during degradation, and the proposed HS-SPME/GC-MS methodology revealed a reliable alternative to the traditionally used distillation techniques.

## Figures and Tables

**Table 1:** Some characteristics of SPME fibers and targeted volatile amines.

Parameters	SPME Fibers			Amines	
	DVB/CAR/PDMS	CAR/PDMS	PDMS/DVB	TMA	DMA
**pKa**	-	-	-	9.80	10.37
**Molecular Weight**	40–275	30–225	50–300	59.11	45.08
**Polarity**	Bipolar	Bipolar	Bipolar	Polar	Polar
**Thickness (µm)**	30–55	75	65	-	-
**Core type**	Stabflex	Fused silica	Fused silica	-	-
**Operating temperature**	230–270	250–320	200–270	-	-
**Extraction mechanism**	Adsorbent	Adsorbent	Adsorbent	-	-

**Table 2:** Method control results.

Method	CMP ^1^	LSL ^2^	USL ^3^	CpK ^4^
HS-SPME	TPA ^5^	0.05	0.17	1.49
	IP ^6^	0.03	0.18	1.47
GC-MS	TPA	0.03	0.18	1.47
	IP	0.02	0.20	1.49
	Resolution	0.04	0.17	1.38
	TF ^7^	0.02	0.19	1.36
	SF ^8^	0.05	0.17	1.37

^1^ CMP-critical method parameters. ^2^ LSL: Lower Specification Limit. ^3^ USL: Upper Specification Limit. ^4^ CpK: Process capability Index. ^5^ TPA: Total Peak Area. ^6^ IP: Intermediate Precision. ^7^ TF: Tailing Factor. ^8^ SF: Symmetry Factor.

**Table 3:** Parameters results of the HS-SPME/GC-MS method for quantification of TMA and DMA in fish.

	TMA	DMA
**Ion (m/z)**	58	44
**Retention time (min)**	4.12	4.23
**Concentration range (µg/mL)**	2-20	2-20
**Regression equation**	Y = 11143x^2^ + 2 × 10^7^ − 1 × 10^7^	Y = 11153x^2^ + 2 × 10^7^ − 3 × 10^7^
***R*^2^**	0.9504	0.9989
**Linearity test (F_theo_/F_exp_)**	3.0 ^1^	15.4 ^1^
**LOD (µg/mL) ^5^**	0.4	0.6
**LOQ (µg/mL) ^6^**	1.3	2.0
Matrix effect (%)	105	109
**Repeatability (%RSD ^7^)**	8.2 ^2^ 6.0 ^3^ 9.6 ^4^	8.3 ^2^ 11.2 ^3^ 7.4 ^3^
**Intermediate Precision (%RSD ^7^)**	8.4 ^2^ 7.1 ^3^ 9.7 ^4^	9.6 ^2^ 13.6 ^3^ 8.8 ^4^
**Recovery (%)**	93 ^2^ 98 ^3^ 99 ^4^	90 ^3^ 93 ^2^ 95 ^4^

^1^*p*-value calculated experimentally through t-Student test between slopes obtained from the calibration curves for TMA in 7.5% TCA and TMA in fish samples. If *p*-value ≥ 0.05, no significant matrix effect was observed. ^2^ LL: low-level concentration (3 µg/mL). ^3^ ML: medium-level concentration (9 µg/mL). ^4^ HL: high-level concentration (15 µg/mL). ^5^ LOD: Limit of detection; ^6^ LOQ: Limit of quantification; ^7^ RSD: Relative standard deviation.

**Table 4:** Estimated rejection day for different assayed methods.

Method	Rejection Criteria	Value at Rejection	Estimated Rejection Day
**QIM**	Off odors	13	7–8
**Torrymeter**	Off odorsSlope change	8	8–9
**Microbiology (TVC)**	Log cfu/cm^2^ = 7–9	8.0	8–9
**Microbiology (H_2_S)**	Log cfu/cm^2^ = 7–9	7.5	9–10
**TMA analysis**	12 mg/100 gr	12.5	9–10

## Supplementary Materials

The following are available online at https://www.mdpi.com/2304-8158/9/9/1321/s1, Figure S1: Ishikawa diagram for quality risk assessment; Figure S2: Pareto chart analysis for HS-SPME and GC-MS; Figure S3: Desirability surface contour method analysis for the effects of all CMPs; Figure S4: The pareto ranking analysis for CG-MS(BP20); Table S1: Chemical methods used for fish freshness determination; Table S2: Gas chromatography works related with TMA and/or DMA extraction; Table S3 - Scheme of the Quality Index Method (QIM) proposed for *Sparus aurata* (GSB); Table S4: Results from DoE 3^4−1^ level, for HS-SPME Knowledge of space, for all tested fibres; Table S5:The HS-SPME/MODR results for the observed vs. predicted values agreement; Table S6: GC-MS MODR results, from DOE fractional factorial design 3^3−1^ level; Table S7: GC-MS/MODR observed vs. predicted values analysis; Table S8: Fractional Factorial Design 3^3−1^ level, for HS-SPME(DVB/CAR/PDMS) robustness analysis; Table S9: GC-MS Robustness results, from DOE fractional factorial design 3^3−1^ level; Table S10: Analysis of agreement between observed and predicted values for HS-SPME robustness; Table S11: GC-MS robustness evaluation by, observed vs. predicted values analysis; Table S12: Attributes evaluation for QIM analysis.
