# Supplementary material for: A Systematic AQbD Approach for Optimization of the Most Influential Experimental Parameters on Analysis of Fish Spoilage-Related Volatile Amines
Source: Foods. 2020 Sep 19;9(9):1321. doi: 10.3390/foods9091321 (PMC7555788; doi:10.3390/foods9091321)
Supplement: Supplementary file 1 [file foods-09-01321-s001.pdf]

A systematic AQbD approach for optimization of experimental most-influencing parameters on analysis of fish spoilage-related volatile amines

Jorge Freitas[a], Pedro Silva[a], Paulo Vaz-Pires[c,d], José S. Câmara[a,e*]

[a] CQM– Centro Química da Madeira, Campus Universitário da Penteada, 9000-039 Funchal, Portugal

[c] ICBAS – Abel Salazar Institute for the Biomedical Sciences, University of Porto, R. Jorge Viterbo Ferreira, 228, 4050-313 Porto, Portugal

[d] CIIMAR – Interdisciplinary Centre of Marine and Environmental Research, Terminal de Cruzeiros de Leixões, Av. General Norton de Matos, S/N, 4450-208 Matosinhos, Portugal

[e] Faculty of Exact Sciences and Engineering, University of Madeira, Campus Universitário da Penteada, 9000-039 Funchal, Portugal

* Corresponding author. Tel. + 351 291 705112; Fax + 351 291 705149

E-mail address: jsc@staff.uma.pt (José S. Câmara)

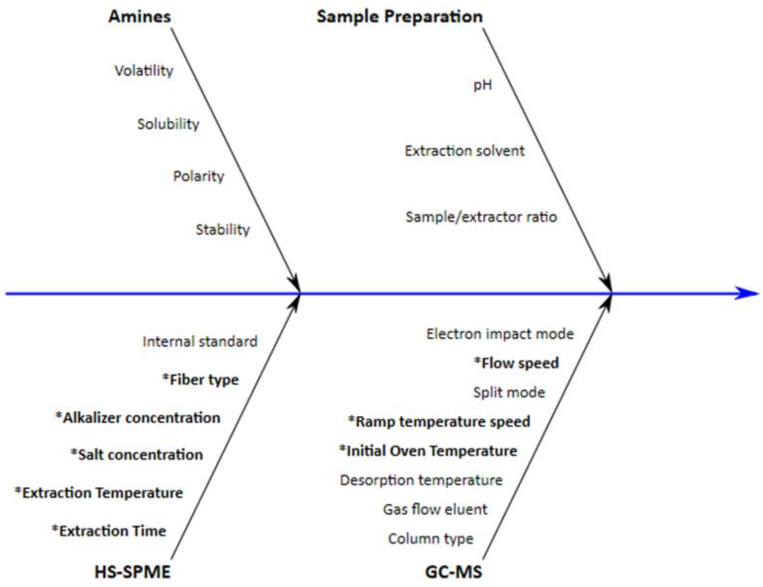

Figure S1. SM. Ishikawa diagram of the selected parameters for quality risk assesSIent (QRA).

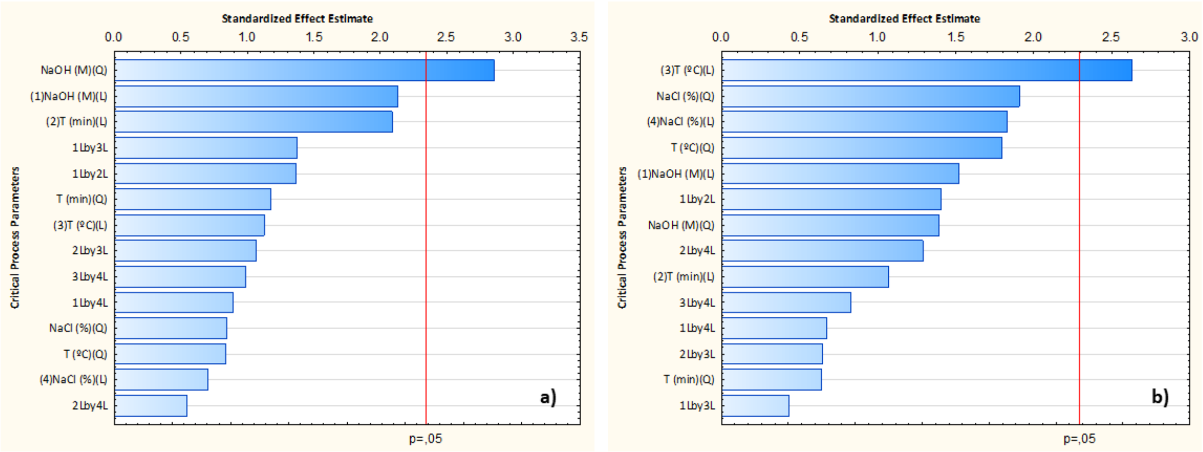

Figure S2. SM - Pareto chart analysis of the effects between the studied CMPs on the TPA and IP response, for HS-SPME$_{(DVB/CAR/PDMS)}$.

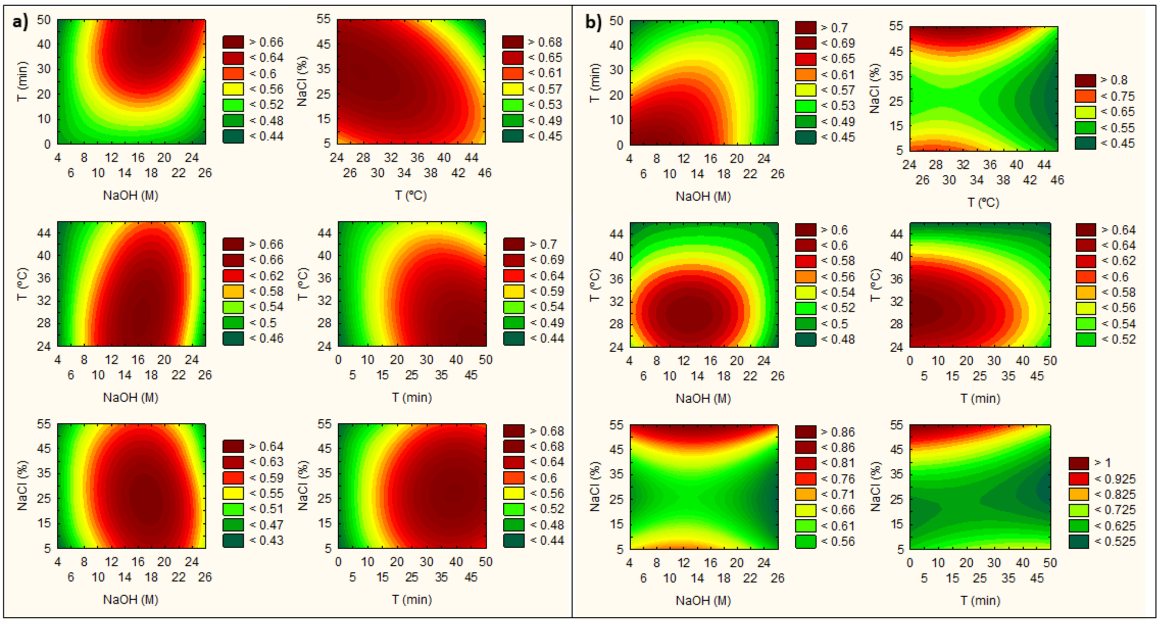

Figure S3. SM Desirability surface contour method analysis. **a)** shows the effects of all CMPs on TPA **b)** shows the effects of all CMPs on IP.

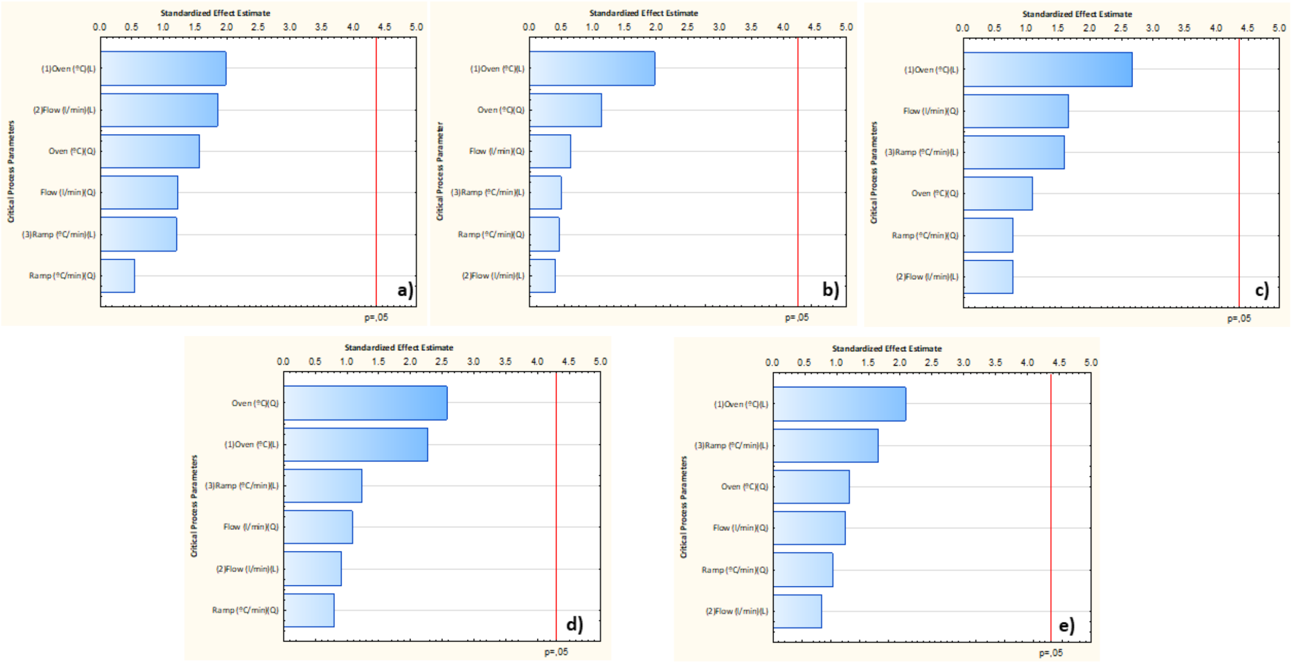

Figure S4.SM The Pareto ranking analysis for CG-MS$_{(BP20)}$. **a)** Total peak area; **b)** Intermediate precision; **c)** Peak resolution; **d)** Tailing factor; **e)** Symmetry factor.

Table S1. SM Chemical methods used for fish freshness determination. Advantages and disadvantages reviewed in Howgate 2012

**Method**	**Advantages**	**Disadvantages**
Direct distillation (L&G)	-	Ammonia formation under unstandardized conditions. Total recovery of DMA is not achieved.
The Picrate Method	-	None of the alkali/formaldehyde combinations completely suppressed the interference of DMA.
Distillation with Formaldehyde	-	The procedure is not specific for TMA. Over estimation of the content (20%). Ammonia is not completely fixed.
Use of muscle extracts	-	Lack of uniformity in calculating the TVB concentration in the muscle tissue. Over estimation (7%) of TVB-N, is common.
Steam distillation (Khejdal)	Semi-automatic and fast.	-
Distillation under reduced pressure	Decomposition of nitrogen-containing substances during direct or steam distillation can be avoided.	Requires a complex apparatus. Time taken to diSlantle and reassemble between analyses.
The Microdiffusion (Conway) Method	Distillation at room temperature.<br>Simplicity of procedure and low cost.	Careful execution for accurate and precise results.<br>Scrupulous cleaning of the cells between analyses.<br>Care needed to avoid air bubbles.
Flow Injection Analysis (FIA)	Distillation at room temperature.<br>Measure the intrinsic TVB content and specific amines.	Practical difficulties with the procedure. Moderately large discrepancies among results between laboratories.
Gas Liquid Chromatography	The analysis of headspace avoids the need for a distillation or extraction step.	Tailing of the amine's pikes. Column packing effect.

Table S2. SM. Gas chromatography works related with TMA and/or DMA extraction with HS-SPME methodology and respective conditions

Gas-Chromatography						HS-SPME				
**Detector**	**GC-column**	**Temperature (℃)**	**Ramp (℃/min)**	**Flow (L/min)**	**Split mode**	**Fiber**	**Extr. Time (min)**	**Extr. Temperature (℃)**	**Alkalizer agent**	**Reference**
MS	DB1 (60 m × 0.32 mm, 5 µm, Agilent)	80/230	15	-	Splitless	DVB/PDMS	10	22	Ammonia	(Wzorek et al., 2010)
MS	Stabilwax DB (30 m × 0.25 mm× 0.25 µm, Restek)	60/210	15	He 1.2	Split 5	CAR/PDMS	35	30	KOH	(Dehaut et al., 2016)
MS	PTA-5 column. (30 m × 0.25 mm i.d.)	35/200	40	He 1.3	Splitless	DVB/CAR/PDMS<br>CW/DVB<br>DVB/PDMS<br>PDMS	5	25	NaOH 15%	(Chan et al., 2006)
FID	Rtx®-VolatileAmine (30 m x 0.32 mm I.D.)	40/120	10	He 1.3	Split 1:25	DVB/CAR/PDMS	15	21	NaOH 15%	(Barbosa-Pereira et al., 2012)
FID	SE-54 (25m × 0.32mm i.d.)	60/230	25	N 12-15	Split 1:100	PDMS-DVB<br>PA<br>Amide bridge-C4	-	-	-	(Li et al., 2004)
FID	-	-	-	He 2.0<br>H 25.0	-	DVB/PDMS	2	35	NaOH 40%	(Béné et al., 2001)

Table S3.SM Scheme of the Quality Index Method (QIM) proposed for *Spaurus aurata* (*GSB*)

**Attribute**	**Parameter**	**Description**	
**Appearance**	Skin	Very bright, iridescent.	0
		Loss of iridescence and bright.	1
		Pale, dull,	2
	Odor	Sea, seaweed, seafood	0
		Neutral	1
		Rancid, metallic	2
		Putrid	3
**Texture**	Firmness	Rigor-mortis	0
		Firm, recovers shape fast (≤ 2 sec.)	1
		Less firm, softness, recovers shape slow (> 2 sec.)	2
		Clearly marked; spongy;	3
**Eyes**	Color	Black; shinny	0
		Black opaque/ slightly milky	1
		Greyish. Distinguishable eyeball.	2
		White/ grey eyeball defined	3
	Shape	Convex	0
		Flat	1
		Concave	2
**Gills**	Color	Dark red	0
		Light red	1
		Brownish red	2
		Brown, grey, discolored, bacteria present	3
	Mucus	Absent	0
		Present, colorless	1
		Whitish/cream	2
		Abundant, brown, yellowish	3
	Odor	Sea, seaweed, seafood	0
		Neutral	1
		Rancid, metallic	2
		Putrid	3
**Total Demerit Points**			**0-22**

Table S4.SM. Results from DoE 3^{4-1} level, for HS-SPME Knowledge of space, for all tested fibres.

Run	Time (min)	NaOH (M)	Temp. (ºC)	NaCl (%)	DVB/CAR/PDMS						CAR/PDMS						PDMS/DVB					
					TMA		DMA		Total		TMA		DMA		Total		TMA		DMA		Total	
					TPA ($x10^7$)	IP (%)	TPA ($x10^7$)	IP (%)	TPA ($x10^7$)	Mean (%)	TPA ($x10^7$)	IP (%)	TPA ($x10^7$)	IP (%)	TPA ($x10^7$)	Mean (%)	TPA ($x10^7$)	IP (%)	TPA ($x10^7$)	IP (%)	TPA ($x10^7$)	Mean (%)
RUN1	5	5	25	10	5.63	1.3	1.69	9.0	7.32	5.1	2.01	1.9	0.49	0.1	2.50	1.0	4.73	13.3	4.93	12.7	5.23	13.0
RUN2	5	5	35	50	8.49	4.2	1.86	0.3	10.3	2.3	2.35	8.7	0.62	9.1	2.97	8.9	4.77	12.2	1.29	9.8	6.07	11.0
RUN3	5	5	45	30	5.25	6.5	2.75	12.2	8.00	9.4	3.42	6.1	0.29	5.7	3.72	5.9	1.36	9.1	0.52	4.6	1.88	6.8
RUN4	5	15	25	50	9.74	3.9	2.45	2.5	12.2	3.2	4.87	10.0	1.31	2.1	6.18	6.0	2.94	14.4	1.56	10.6	4.51	12.5
RUN5	5	15	35	30	8.16	5.0	6.07	6.7	14.2	5.8	1.91	9.6	0.88	12.6	2.79	11.1	2.93	10.8	2.04	14.8	4.97	12.8
RUN6	5	15	45	10	8.80	11.0	8.18	5.7	17.0	8.3	5.10	2.9	1.78	11.8	6.88	7.4	1.56	11.5	1.66	13.8	3.23	12.7
RUN7	5	25	25	30	5.59	6.9	1.43	6.9	7.02	6.9	1.74	10.8	0.65	6.6	2.39	8.7	5.55	12.6	3.79	9.6	9.35	11.1
RUN8	5	25	35	10	6.46	12.5	3.41	5.0	9.86	8.7	3.60	8.0	1.38	10.4	4.98	9.2	1.52	9.5	1.90	12.6	3.42	11.0
RUN9	5	25	45	50	6.90	12.5	6.89	4.8	13.8	8.6	2.58	10.0	2.52	13.4	5.10	11.7	3.58	4.5	3.17	14.0	6.74	9.3
RUN10	25	5	25	50	7.54	2.4	3.12	8.1	10.7	5.2	3.60	3.8	1.54	11.0	5.14	7.4	2.71	13.8	2.59	7.9	5.29	10.8
RUN11	25	5	35	30	12.4	1.5	5.88	6.6	18.2	4.1	2.13	2.9	1.54	6.7	3.68	4.8	2.57	1.4	2.73	1.0	5.30	1.2
RUN12	25	5	45	10	1.95	7.7	2.28	12.2	4.23	9.9	1.16	11.7	0.74	3.1	1.90	7.4	2.07	0.7	2.59	11.2	4.67	5.9
RUN13	25	15	25	30	21.6	10.2	10.9	4.7	32.4	7.4	3.92	7.3	2.26	11.1	6.19	9.2	5.39	12.4	3.30	2.8	8.69	7.6
RUN14	25	15	35	10	15.0	5.4	8.87	6.8	23.9	6.1	2.83	0.8	1.15	3.6	3.97	2.2	0.82	6.5	0.67	0.0	1.49	3.3
RUN15	25	15	45	50	3.66	1.9	4.33	1.5	8.00	1.7	15.8	7.8	5.25	13.1	21.1	10.5	4.88	12.3	4.03	4.7	8.91	8.5
RUN16	25	25	25	10	11.3	8.2	5.20	7.2	16.5	7.7	9.84	8.0	6.83	4.6	16.7	6.3	3.69	15.4	3.12	9.8	6.80	12.6
RUN17	25	25	35	50	9.74	6.4	6.79	10.4	16.5	8.4	6.25	6.5	4.50	11.5	10.8	9.0	6.39	6.9	5.13	11.3	11.5	9.1
RUN18	25	25	45	30	8.15	3.6	6.88	15.0	15.0	9.3	15.3	5.9	7.58	9.5	22.9	7.7	2.47	11.0	2.76	5.6	5.22	8.3
RUN19	45	5	25	30	7.95	5.7	4.49	10.7	12.4	8.2	3.99	11.9	1.61	8.4	5.60	10.1	3.91	1.2	1.93	0.4	5.84	0.8
RUN20	45	5	35	10	4.20	4.9	3.63	3.2	7.83	4.1	0.98	11.2	0.79	1.8	1.78	6.5	2.10	0.9	2.46	11.0	4.56	6.0
RUN21	45	5	45	50	2.45	10.8	2.34	9.2	4.78	10.0	10.4	4.4	4.85	0.6	15.3	2.5	1.10	12.0	1.49	12.2	2.59	12.1
RUN22	45	15	25	10	9.85	6.3	10.2	4.3	20.0	5.3	7.59	5.9	3.21	2.2	10.8	4.1	6.60	5.9	5.15	1.7	11.7	3.8
RUN23	45	15	35	50	18.2	6.5	9.01	3.2	27.2	4.9	7.79	12.6	5.56	12.5	13.3	12.5	4.68	4.8	3.35	9.8	8.03	7.3
RUN24	45	15	45	30	11.9	14.3	8.94	10.1	20.9	12.2	18.9	8.1	7.02	5.3	26.0	6.7	2.28	10.2	2.20	6.8	4.48	8.5
RUN25	45	25	25	50	12.2	4.9	8.58	3.2	20.8	4.0	9.31	0.1	5.47	5.8	14.8	2.9	2.81	6.4	3.18	10.4	5.99	8.4
RUN26	45	25	35	30	4.60	6.7	6.66	9.2	11.3	7.9	8.84	5.6	4.67	12.8	13.5	9.2	2.36	3.3	2.78	7.2	5.13	5.2
RUN27	45	25	45	10	14.9	3.8	11.2	14.7	26.1	9.2	18.4	6.1	1.94	11.7	20.3	8.9	1.82	12.9	1.27	3.5	3.09	8.2

Table S5.SM The HS-SPME/MODR results for the observed vs predicted values agreement.

	Total Peak Area (x10^{8})							Intermediate Precision (% RSD)			
	**Observed 1**	**Observed 2**	**Observed 3**	**Predicted**	**C.I. -95%**	**C.I. + 95%**		**Observed**	**Predicted**	**C.I. -95%**	**C.I. + 95%**
**TMA**	2.31	2.00	2.16	1.47	0.88	2.07	**TMA**	10.2	5.2	0.9	9.6
**DMA**	1.13	1.05	1.09	0.79	0.55	1.04	**DMA**	4.7	7.0	3.3	10.8
**Sum**	3.44	3.05	3.24	2.26	1.45	3.07	**Mean**	7.4	6.1	3.2	9.1

Table S6.SM GC-MS MODR results, from DOE fractional factorial design 3^{3-1} level.

Run	Oven (ºC)	Flow (L/min)	Ramp (ºC/min)	TMA					DMA					Total				
				TPA ($x10^7$)	IP (%)	PR	TF	SF.	TPA ($x10^7$)	IP (%)	PR	TF	SF.	TPA ($x10^7$)	IP (%)	PR	TF	SF
RUN1	35	0.8	40	1.48	9.9	7.0	0.77	0.50	6.82	13.7	3.3	0.58	2.13	8.29	11.8	5.17	0.68	1.32
RUN2	35	1.0	60	1.71	13.8	9.2	0.81	0.60	1.48	15.0	26.1	0.07	0.21	3.20	14.4	17.6	0.44	0.41
RUN3	35	1.3	50	2.36	9.3	13.1	0.72	0.35	13.8	12.4	3.7	0.54	2.11	16.2	10.8	8.39	0.63	1.23
RUN4	60	0.8	60	1.62	1.6	4.8	0.93	0.85	5.73	14.5	4.0	0.73	0.33	7.35	8.1	4.41	0.83	0.59
RUN5	60	1.0	50	2.15	12.5	4.7	0.94	0.82	6.07	9.4	4.3	0.74	0.36	8.22	10.9	4.50	0.84	0.59
RUN6	60	1.3	40	2.02	8.2	4.8	0.99	0.86	5.64	6.6	4.9	0.76	0.38	7.66	7.4	4.83	0.87	0.62
RUN7	85	0.8	50	5.05	11.1	3.2	0.84	0.59	7.44	10.3	2.2	0.63	0.16	12.5	10.7	2.69	0.74	0.38
RUN8	85	1.0	40	6.44	7.7	3.0	0.92	0.75	7.57	8.0	2.2	0.60	2.15	14.0	7.8	2.64	0.76	1.45
RUN9	85	1.3	60	17.6	8.4	2.9	0.80	0.47	19.3	8.9	2.1	0.58	0.04	36.9	8.6	2.53	0.69	0.25

Table S7.SM GC-MS /MODR observed vs predicted values analysis.

	Observed	Predicted	C.I. -95%	C.I. + 95%
**TPA**	1.40E+08	1.42E+08	-1.87E+08	4.71E+08
**IP**	7.83	8.99	2.28	15.69
**PR**	2.64	3.37	-11.46	18.19
**TF**	0.72	0.70	0.40	1.00
**RS**	0.57	0.40	-0.99	1.80

Table S8.SM Fractional Factorial Design 3^{3-1} level, for HS-SPME$_{(DVB/CAR/PDMS)}$ robustness analysis.

**Run**	**Temperature (ºC)**	**NaOH (M)**	**Time (min)**	**TMA ($x10^8$)**	**IP (%)**	**DMA ($x10^8$)**	**IP (%)**	**Total ($x10^8$)**	**Mean (%)**
Run1	33.0	14.0	28.0	1.73	11.9	3.86	10.0	5.59	11.0
Run2	33.0	15.0	32.0	2.42	9.2	4.35	5.6	6.77	7.4
Run3	33.0	16.0	30.0	2.19	7.3	4.83	4.0	7.02	5.7
Run4	35.0	14.0	32.0	2.34	7.6	3.51	10.6	5.85	9.1
Run5	35.0	15.0	30.0	2.34	2.3	4.71	1.1	7.04	1.7
Run6	35.0	16.0	28.0	2.43	4.6	4.52	9.6	6.95	7.1
Run7	37.0	14.0	30.0	2.26	8.0	4.61	9.4	6.87	8.7
Run8	37.0	15.0	28.0	1.80	5.8	3.56	7.0	5.35	6.4
Run9	37.0	16.0	32.0	1.69	11.4	3.51	8.6	5.20	10.0

Table S9.SM GC-MS Robustness results, from DOE fractional factorial design 3^{3-1} level

Run	Oven (ºC)	Flow (L/min)	Ramp (ºC/min)	TMA					DMA					Total				
				TPA ($x10^8$)	IP (%)	PR	TF	SF.	TPA ($x10^8$)	IP (%)	PR	TF	SF.	TPA ($x10^8$)	IP (%)	PR	TF	SF
RUN1	75	0.8	45	2.64	6.5	2.9	0.83	2.63	3.93	5.3	1.9	0.59	2.27	6.57	5.9	2.4	0.71	2.45
RUN2	75	1	55	2.32	2.1	2.2	0.74	2.61	4.88	3.0	2.2	0.56	2.23	7.19	2.5	2.2	0.65	2.42
RUN3	75	1.2	50	1.82	1.2	3.0	0.76	2.56	3.58	2.7	3.0	0.55	2.22	5.40	2.0	3.0	0.66	2.39
RUN4	80	0.8	55	2.64	6.4	2.0	0.76	2.48	4.83	5.0	2.0	0.58	2.26	7.47	5.7	2.0	0.67	2.37
RUN5	80	1	50	2.19	3.4	2.3	0.78	2.55	3.80	5.7	2.3	0.56	2.24	5.99	4.6	2.3	0.67	2.39
RUN6	80	1.2	45	2.12	8.7	2.5	0.76	2.57	3.97	4.7	2.5	0.55	2.23	6.09	6.7	2.5	0.66	2.40
RUN7	85	0.8	50	2.75	3.3	1.7	0.81	2.77	4.67	15.6	1.7	0.58	2.26	7.42	9.5	1.7	0.70	2.52
RUN8	85	1	45	2.58	7.6	2.0	0.76	2.53	3.79	9.3	2.0	0.55	2.23	6.36	8.4	2.0	0.66	2.38
RUN9	85	1.2	55	1.88	2.9	2.2	0.79	2.81	3.47	4.5	2.2	0.56	2.23	5.36	3.7	2.2	0.68	2.52

Table S10.SM Analysis of agreement between observed and predicted values for HS-SPME robustness.

	Total Peak Area ($x10^8$)					Intermediate Precision (% RSD)			
	**Observed**	**Predicted**	***C.I. -95%**	***C.I. + 95%**		**Observed**	**Predicted**	***C.I. -95%**	***C.I. + 95%**
**TMA**	2.34	2.55	1.06	4.04	**TMA**	2.3	1.3	-4.3	6.9
**DMA**	4.71	4.84	2.95	6.74	**DMA**	1.1	1.9	-3.6	7.3
**Total**	7.04	7.39	4.12	10.7	**Mean**	1.7	1.6	-0.7	3.9

***C.I.** – Confidence Interval

Table S11.SM GC-MS robustness evaluation by, observed vs predicted values analysis.

	Observed	Predicted	C.I. -95%	C.I. + 95%
**TPA**	5.99E+08	6.45E+08	3.39E+08	9.51E+08
**IP**	4.56	5.28	0.31	10.2
**PR**	2.27	2.21	1.48	2.94
**TF**	0.67	0.66	0.58	0.74
**RS**	2.39	2.36	2.10	2.63

Table S12.SM Attributes evaluation for QIM analysis.

Days in ice	Skin	Odor	Anus	Firmness	Eye color	Eye shape	Gill color	Gill Mucus	Gill Odor	Q.I
**0**	0.0	0.0	0.0	0.0	0.0	0.0	0.5	0.0	0.0	0.5
**1**	0.2	0.2	0.3	0.3	0.0	0.2	0.2	0.2	0.5	2.1
**3**	0.8	1.0	0.0	1.0	0.5	0.5	1.3	0.8	1.0	6.8
**5**	1.5	1.2	0.0	1.0	1.0	1.2	1.5	1.3	1.2	9.8
**7**	1.7	1.4	1.2	1.6	1.1	1.5	1.8	1.4	1.6	13.2
**9**	2.0	1.0	0.0	2.0	2.0	2.0	3.0	2.0	2.0	16.0
**12**	2.0	2.0	1.0	2.0	2.0	2.0	2.5	2.5	3.0	19.0
**14**	2.1	2.3	2.2	2.3	2.2	2.3	2.9	2.8	2.9	21.9
